# Signaling pathways and targeted therapy for rosacea

**DOI:** 10.3389/fimmu.2024.1367994

**Published:** 2024-09-16

**Authors:** Fengjuan Yang, Lian Wang, Deyu Song, Lu Zhang, Xiaoyun Wang, Dan Du, Xian Jiang

**Affiliations:** ^1^ Department of Dermatology, West China Hospital, Sichuan University, Chengdu, China; ^2^ Laboratory of Dermatology, Clinical Institute of Inflammation and Immunology, Frontiers Science Center for Disease-Related Molecular Network, West China Hospital, Sichuan University, Chengdu, China

**Keywords:** rosacea, pathogenesis, signaling pathways, targeted therapy, review

## Abstract

Rosacea is a chronic skin inflammatory disease with a global prevalence ranging from 1% to 20%. It is characterized by facial erythema, telangiectasia, papules, pustules, and ocular manifestations. Its pathogenesis involves a complex interplay of genetic, environmental, immune, microbial, and neurovascular factors. Recent studies have advanced our understanding of its molecular basis, focusing on toll-like receptor (TLR) 2 pathways, LL37 expression, mammalian target of rapamycin (mTOR) activation, interleukin (IL)-17 signaling, transient receptor potential vanilloid (TRPV) functions, and the Janus kinase-signal transducer and activator of transcription (JAK-STAT) pathways. LL37-associated signaling pathways, particularly involving TLR2 and mTORC1, are critical in the pathogenesis of rosacea. LL37 interacts with signaling molecules such as extracellular signal-regulated kinases 1 and 2 (ERK1/2), nuclear factor kappa B (NF-*κ*B), inflammasomes, C-X-C motif chemokine ligand 8 (CXCL8), mas-related G-protein-coupled receptor X2 (MRGPRX2)-TRPV4, and vascular endothelial growth factor (VEGF). This interaction activates macrophages, neutrophils, mast cells, and vascular endothelial cells, leading to cytokine release including tumor necrosis factor-alpha (TNF-*α*), IL-6, IL-1*β*, C motif chemokine ligand (CCL) 5, CXCL9, and CXCL10. These processes contribute to immune response modulation, inflammation, and angiogenesis in rosacea pathophysiology. The IL-17 signaling pathway also plays a crucial role in rosacea, affecting angiogenesis and the production of inflammatory cytokines. In addition, recent insights into the JAK/STAT pathways have revealed their integral role in inflammatory and angiogenic mechanisms associated with rosacea. Rosacea treatment currently focuses on symptom management, with emerging insights into these molecular pathways providing more targeted and effective therapies. Biological agents targeting specific cytokines, IL-17 inhibitors, JAK inhibitors, and VEGF antagonists are promising for future rosacea therapy, aiming for enhanced efficacy and fewer side effects. This review provides a comprehensive overview of the current knowledge regarding signaling pathways in rosacea and potential targeted therapeutic strategies.

## 1 Introduction

Rosacea is a common chronic skin inflammatory disease affecting 1% to 20% of the global population (1). It is characterized by various signs and symptoms, including erythema, telangiectasia, papules, pustules, and flushing with burning and stinging sensations on the central face (2). Rosacea is categorized into four subtypes: erythematotelangiectatic rosacea (ETR), characterized by persistent erythema and telangiectasia on the central face; papulopustular rosacea (PPR), presenting with persistent facial erythema, papules, and pustules; phymatids rosacea (PhR), marked by thickened skin and an irregular surface texture; and ocular rosacea (3). A key hallmark of rosacea is its hypersensitivity to various stimuli like temperature changes, ultraviolet light (UV), emotional changes, and certain foods such as spicy food (4). Rosacea often impacts the facial area, significantly affecting patients’ self-esteem and mental health, and is associated with systemic diseases like hypertension, inflammatory bowel disease, autoimmune disorders, and migraines (5).

Current research indicates that the pathogenesis of rosacea is mainly due to the cross-talk of genetic and environmental factors (4, 6). This includes immune dysfunction, chronic inflammation, microbial imbalances, and vascular neurologic dysfunction (7). Recent molecular studies have identified critical signaling pathways in rosacea, highlighting the roles of toll-like receptor (TLR)2, LL37 production (8), the interleukin (IL)-17 signaling pathway (9), and the LL37- mammalian target of rapamycin (mTOR) and Janus kinase-signal transducer and activator of transcription (JAK-STAT) pathways (10, 11). These discoveries are crucial for developing targeted treatments. Currently, the treatments of rosacea are primarily symptombased, with effective solutions still under research (12). This review provides a detailed understanding of the signaling pathways involved in rosacea, as well as the emerging targeted therapeutic strategies.

## 2 LL37-related signaling pathways

TLRs play a crucial role in recognizing pathogen-associated molecular patterns (PAMPs) and damage-associated molecular patterns (DAMPs) (13), triggering anti-pathogen responses, including antimicrobial peptide secretion and proinflammatory cytokine and chemokine production (14). TLR2, a primary pattern recognition receptor, is significantly overexpressed in rosacea patients’ keratinocytes, contributing to heightened skin sensitivity to various stimuli (15). TLR2 is also expressed in sensory neurons, and the TLR2 signaling pathway contributes to the mechanism of neurological dysfunction in rosacea (16). Numerous studies have confirmed that TLR2 responds to environmental stimuli such as reactive oxygen species (ROS), microbial imbalance, Demodex mites, UVB radiation, and temperature changes (17–19). Glucocorticoids can increase TLR2 expression in epidermal keratinocytes, potentially leading to glucocorticoid-induced rosacea-like dermatitis (20). And these trigger factors can amplify TLR2 expression through enhanced endoplasmic reticulum (ER) stress and activating transcription factor 4 (ATF4) upregulation (16). Upon TLR2 activation, Kallikrein 5 (KLK5) and total serine protease activity are released from keratinocytes, a process reduced by TLR2-deficient mice. TLR2’s ability to release KLK5 is calcium-dependent, with TLR2 ligands triggering a calcium influx that increases KLK5 release (15, 21). KLK5 is also mediated by Metalloproteinases (MMPs), which decompose the extracellular matrix (22). MMP2 and MMP9 are associated with the pathogenesis of rosacea, with elevated MMP-9 mRNA levels in rosacea patients’ facial skin (17, 23, 24).

**Figure 1:**
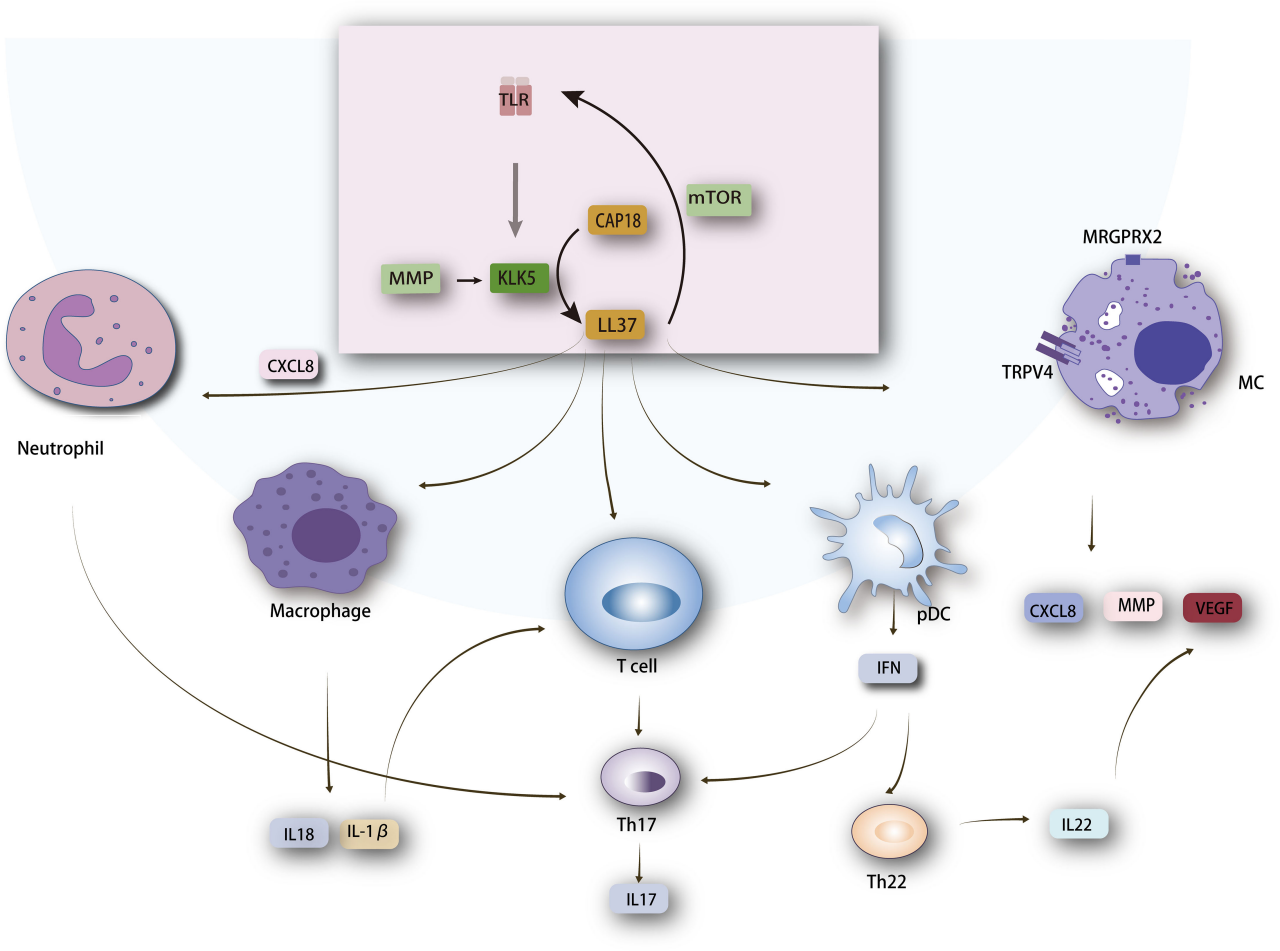
Mechanism of LL37 in Rosacea Pathogenesis. LL37 interacts with several key molecules, including Toll-like receptor 2 (TLR2), mechanistic target of rapamycin complex 1 (mTORC1), chemokine (C-X-C motif) ligand 8 (CXCL8), and Mas-related G-protein coupled receptor member X2 (MRGPRX2) linked to transient receptor potential vanilloid 4 (TRPV4). These interactions lead to the activation of various cell types such as macrophages, neutrophils, T cells, mast cells, and plasmacytoid dendritic cells (pDCs). Activation of these cells results in the production of cytokines, playing a critical role in inflammation, immune modulation, and angiogenesis in rosacea. The cytokines produced, such as IL-1*β* and TNF-*α*, contribute to the inflammatory responses characteristic of rosacea. The figure showcases the crucial LL37-mediated pathways and their roles in the pathogenesis of rosacea, emphasizing the complex interplay between different cell types and signaling molecules.

Cathelicidin, an antimicrobial peptide (AMP), acts as an endogenous antibiotic (25). It is initially inactive and activated by serine proteases into multiple active peptides. Specifically, KLK5, a trypsin-like serine protease, is key in converting cathelicidin into LL37 by processing its precursor, hCAP18 (human cationic antimicrobial protein of 18 kDa) (26). Research by Mylonas A. et al. have revealed that KLK5 cleaves cathelicidin, producing peptides with increased DNA binding and enhanced induction of type I interferons (IFNs) in plasmacytoid dendritic cells (pDCs) (27). Cathelicidin expression is regulated by vitamin D–dependent mechanisms involving the vitamin D receptor, controlling human cathelicidin in various cell types, as well as vitamin D-independent mechanisms that increase cathelicidin expression in response to external stressors like infections, injuries, or barrier disruption, often coinciding with ER stress (28–30). LL37 is produced via the TLR2-KLK5 pathway in response to stimuli such as temperature increase. Moreover, mTORC1, a serine/threonine protein kinase, regulates cathelicidin expression in keratinocytes through a positive feedback mechanism. LL37 binds to TLR2, activating mTORC1 signaling and increasing LL37 expression in keratinocytes, highlighting mTORC1’s vital role in LL37 amplification (10, 31, 32). LL37 is central to rosacea pathogenesis, being overexpressed in rosacea patients’ lesional skin (33–35). Intradermal injection of human LL37 in mice models induces inflammatory responses similar to rosacea, making it a key model in rosacea research (36, 37). LL37 has multiple functions, including immune response modulation, inflammation, and angiogenesis (33, 38). It activates mast cells (MCs), keratinocytes, neutrophils, and macrophages, leading to pro-inflammatory cytokine production, leukocyte chemotaxis, MMP expression, and angiogenesis (36, 39–42). LL37-associated signaling pathways are shown in **Figure 1**.

### 2.1 LL37- MRGPRX2-TRPV4 pathway in rosacea

LL-37, a potent chemoattractant, activates MCs in the inflammatory cascades. Increased MC concentration and degranulation, with a positive correlation between MC density and rosacea duration (43). In MC-deficient mice, rosacea-like symptoms are absent following LL37 dermal injection (44, 45). Subramanian H. et al. identified LL37’s induction of MCs through the Mas-related G-protein-coupled receptor-X2 (MRGPRX2) (46). *β*-arrestin 2 (*β*arr2) regulates this via extracellular Signal-Regulated Kinase 1 and 2 (ERK1/2) phosphorylation and nuclear factor kappa B (NF-*κ*B) activation in mice, suggesting potential therapeutic targets in rosacea (47). Sulk M. et al. observed an upregulation of the transient receptor potential vanilloid (TRPV) 4 channel, co-localized with MCs in rosacea patients (48). LL37 directly increases TRPV4 expression in human MCs via MRGPRX2. This elevation in TRPV4 likely facilitates greater cation influx, raising intracellular Ca2+ levels and priming MCs for continuous degranulation or transgranulation (49, 50). Activated MCs release various cytokines, including IL-1, transforming growth factor (TGF-*β*), tumor Necrosis Factor-alpha (TNF-*α*), and vascular endothelial growth factor (VEGF) (51). Additionally, MMP9 mRNA, a key MC marker, is upregulated in rosacea-affected skin, primarily near blood vessels (45). Neutrophils, which are recruited following mast cell (MC) activation, are a significant source of LL-37. This creates a feedback loop that perpetuates MC activation and chronic cutaneous inflammation in rosacea (52). Therefore, MCs are crucial in cathelicidin-induced skin inflammation through their role in cytokine and bioactive mediator secretion upon stimulation (53).

### 2.2 LL37-IL1*β*/IL17 pathway in rosacea

The NF-*κ*B and the mitogen-associated protein kinase (MAPK) signaling pathway are crucial in LL37mediated inflammation (54, 55). LL-37 activates MAPK, leading to phosphorylation of ERK1/2 and p38 kinases (56), and induces NF-*κ*B-mediated gene expression (57, 58). These pathways play a central role in the pathogenesis of rosacea, as evidenced by increased p38 and ERK levels in ocular rosacea tissue (59), upregulated MAPK pathways in PPR lesional tissue (60), and elevated NF-*κ*B activity in rosacea patients’ eyelid samples (61). Furthermore, TLR signaling pathways also converge on MAPK and NF*κ*B-dependent gene expression (62). Importantly, the TLR2/Myeloid differentiation factor-88 adaptor protein (MyD88)/NF-*κ*B is implicated in rosacea pathogenesis, as suggested by elevated MyD88 levels in rosacea skin biopsies (63). Moreover, dietary supplementation with n-3 PUFAs has been shown to ameliorate skin inflammation in an experimental rosacea model by inhibiting this pathway (64). Deng Z. et al. noted that LL37 initiates NF-*κ*B activation, possibly through mTORC1 signaling (10). Additionally, UV radiation-induced ROS in keratinocytes activates MAPK and NF-*κ*B pathways, influencing inflammatory signaling (65, 66). These pathways control inflammatory cytokine gene expression in immune cells (67, 68). Specifically, the expression of two NF-*κ*B target genes, namely IL-1*α* and IL-1*β*, was elevated in rosacea (60, 69).

LL-37 also enhances the ability to release IL-1*β* by activating the inflammasome (70). NLRP3 (NOD-, LRR- and pyrin domain-containing protein 3) deficiency reduces LL37-induced rosacea-like inflammation (39). NLRP3, an intracellular sensor, is overexpressed in PPR subjects (71). The formation of the NLRP3 inflammasome subsequently leads to the caspase 1-dependent release of the pro-inflammatory cytokines IL-1*β* and IL-18 (72). IL-1*β* emerges as a critical mediator in the inflammation development in PPR (60). IL-18, an integral constituent of the IL-1 cytokine family, is heightened in rosacea patients (73). TNF-*α* signaling also upregulates IL-1*β* expression (60).

IL-1*β* serves as a co-stimulator of the proliferation of T-cells and is linked to Th17 lymphocyte differentiation (74). Th17 cells, active in rosacea, release proinflammatory cytokines, prominently IL-17. In rosacea, T-cell-dominated lymphocytes infiltrate affected skin (75), with consistently elevated IL-17 serum levels (76). Thus, IL-17 plays a crucial role in rosacea pathogenesis, particularly in PPR (77, 78). IL-17 has diverse functions. It activates VEGF-induced angiogenesis and expansion, as shown in both *in vitro* and *in vivo* studies (79). Obradovic´ H. et al. found that recombinant mouse IL-17 induces MMP9 expression in mouse myoblast C2C12 cells after IL-17 treatment (80).Furthermore, IL-17 stimulates vitamin-D3-induced LL37 production in keratinocytes (81, 82). Remarkably, LL37 induces genes related to Th1/Th17 polarization (83). IL-17 also prompts the production of pro-inflammatory cytokines, including TNF-*α*, IL-1*β*, IL-8, and IL-6 (84). Rosacea skin samples show increased expression of these cytokines (85). Apart from Th17 cells, Th1 cells are also involved in the pathogenesis of rosacea. Th1 cells secrete IFN-*γ*, a potent macrophage activator that classically activates human macrophages into a pro-inflammatory (M1) phenotype *in vitro (* 86). This enhances the interaction between CD4+ T cells and the innate immune system in the disease.

### 2.3 LL37-CXCL8 interaction in rosacea

LL37 induces the release of C-X-C motif Chemokine ligand (CXCL) 8 (formerly known as IL-8) from keratinocytes, a crucial chemotactic factor for neutrophils in rosacea (57, 87). Transcriptome analyses showed increased CXCL8 expression in rosacea (88, 89). Neutrophil migration is prompted by Demodex folliculorum and its associated bacillus oleronius in rosacea (90). These neutrophil pathways and proteins are central to rosacea’s inflammation, with pustule development indicative of neutrophil infiltration (88). Neutrophils play a vital role in microbial defense, neutralizing threats through enzyme release, ROS synthesis, and inflammatory mediator production (91). This influx of neutrophils, in turn, precipitates the secretion of IL-17, thereby establishing a chronic inflammation cycle in rosacea.

### 2.4 LL37-VEGF axis in rosacea pathogenesis

Angiogenesis, facilitated by VEGF, is central to rosacea’s hallmark symptoms of flushing and erythema (92). VEGF serves dual roles in angiogenesis and inflammation (93). In facial redness, VEGF, VEGF-R1, and VEGF-R2 are upregulated in the granular layer and stratum corneum of keratinocytes, as well as in dermal leukocytes including lymphocytes, macrophages, and plasma cells (94, 95). The VEGF polymorphism (+405C/G) is linked to rosacea severity (96). CD31+ cells infiltrates are primary sources of VEGF, driving angiogenesis (97). VEGF production by activated T cells stimulates angiogenesis and promotes Th1 cell differentiation, creating a feedback loop (98, 99). Additionally, UVB exposure activates VEGF signaling, with VEGF-A intensifying vascular sensitivity to UVB (100).

LL37 contributes to angiogenesis in rosacea. It activates endothelial cells (ECs) and VEGF via FPRL1, promoting angiogenesis (101). mTORC1 signaling mediates LL37-induced angiogenesis, with activation noted in ECs of rosacea lesions and LL37-induced rosacea-like mouse models (102). Furthermore, LL37-induced type I IFNs from pDCs, overexpressed during rosacea flare-ups, lead to an increased Th22/Th17 cytokine response (27). Enhanced IL-22 expression and EC sensitization to IL-22 facilitate aberrant angiogenesis (27). Moreover, TLR2 pathway overexpression in keratinocytes augments proinflammatory cytokine and chemokine expression, including IL-8, IL-1*β*, TNF-*α*, and C motif chemokine ligand (CCL) 5, CXCL9, CXCL10, and CXCL11 (8). These elevated levels of cytokines and chemokines result in the induction of vascular hyper-reactivity (103).

A recent study investigated the role of Hippo signaling pathway, specifically yes-associated protein (YAP) and transcriptional coactivator with PDZ-binding motif (TAZ), in rosacea. The study found alterations in these signaling molecules in rosacea patients, suggesting their involvement in the development of new angiogenesis within the skin. Furthermore, the study showed that inhibiting YAP/TAZ reduced VEGF immunoreactivity, a marker of blood vessel formation. These findings suggest that YAP/TAZ may play a role in the mechanisms by which rosacea causes abnormal blood vessel growth (104).

## 3 JAK/STAT signaling pathway

The JAK/STAT pathways have a wide range of functions on immune responses, cellular proliferation, differentiation, apoptosis, and immunoregulation (105). JAK inhibitors are increasingly used in treating inflammatory skin disorders (106). In LL37-treated HaCaT cells, elevated JAK2 and STAT3 levels suggest a strong connection between JAK/STAT signaling and rosacea’s inflammatory response. JAK2/STAT3 activation interacts with TLR2 signaling (107), leading to increased production of pro-inflammatory cytokines like TNF-*α*, IL-6, and IL-8 (108, 109).

Rosacea’s inflammation and immune infiltration are exacerbated by skin barrier disruption, partly due to STAT3-mediated cytokine signaling in keratinocytes (110). STAT3 also regulates degranulation in human and mouse MCs (111, 112). ERK1/2-mediated mitochondrial STAT3 phosphorylation contributes to MC degranulation (113). Blazanin N.et al. observed that acute solar UV exposure activates pSTAT1-related signaling in keratinocytes (114), indicating epidermal-derived STAT1’s role in epithelial-immune communication in rosacea (115). The role of IL-17 in increasing VEGF expression via JAK/STAT signaling has been demonstrated in various contexts. IL-17 has been shown to induce reactive astrocytes and upregulate VEGF through JAK/STAT signaling, as well as up-regulate VEGF in nucleus pulposus cells via the same pathway. These findings suggest that similar mechanisms might be relevant to the inflammatory response in rosacea (116, 117).

## 4 Cutaneous neuroinflammation and downstream signal pathways in rosacea

Cutaneous neurogenic inflammation (CNI) is widely recognized in rosacea, involving a series of signaling cascades. Ion channels, particularly transient receptor potential (TRP) channels in skin nerve fibers, activate upon stimuli, releasing vasoactive neuropeptides that interact with keratinocytes, immune cells, and blood vessels (118, 119). These neuropeptides exacerbate inflammation and vascular dilation, translating nerve impulses into signals for immune cells. Rosacea is a classic example of CNI, which can be explained by the neurologic hypersensitivity in patients with rosacea (120).

TRPV1, a critical cation channel primarily for Ca2+, is involved in cutaneous neurogenic inflammation and pain (121). TRPV1 expression increases in rosacea, especially in keratinocytes (122), upon stimulation by factors like pH changes, high temperatures, and UVB exposure (123, 124). Activated TRPV1 stimulates sensory neuron C fibers, releasing mediators that contribute to neurogenic inflammation and pain through elevated cytosolic Ca2+ levels. This leads to increased release of neuropeptides such as pituitary adenylate cyclase-activating polypeptide (PACAP), vasoactive intestinal peptide (VIP), VEGF, adrenomedullin, calcitonin gene-related peptide (CGRP), and substance P (SP), all implicated in rosacea pathogenesis (125–127). These neuropeptides collaborate in processes like inflammation, tissue damage, vasomotor disturbances, and increased neurovascular reactivity (128, 129). Abnormal amino acid metabolism, specifically glutamic and aspartic acids, can enhance the formation of erythema and telangiectasia in rosacea-like mouse skin through vasodilatory neuropeptides in peripheral neurons and keratinocytes (130).

CGRP, a potent microvascular dilator, contributes to extensive neurogenic vasodilation and mobilizes inflammatory cells (127). It also modulates cutaneous immunity by affecting NF-*κ*B expression in immune cells (131). SP influences the emergence of edema in rosacea through its interaction with neurokinin 1 receptors and contributes to MCs degranulation, EC proliferation, and localized vasodilation (118, 132). Intradermal PACAP38 administration increases pain perception and skin blood flow, exacerbating rosacea features like facial flushing and edema (125). Mechanistically, PACAP acts as a potent vasodilator and influences vascular responses in human skin (133). It upregulates MC proteases (MMP-1 and MMP-9) and proinflammatory cytokines, including TNF and CXCL2, and may affect the pathway converting hCAP18 into LL37 (134). VIP enhances Th17 cell differentiation, shifting the T-helper cell response towards Th17 (135). In brief, VIP, PACAP, and CGRP act as vasodilators and mediate the production of inflammatory factors through interaction with skin immune cells.

Furthermore, there is notable upregulation of TRPV expression in rosacea, affecting not only neuronal but also non-neuronal cells. Sulk M.et al. observed increased dermal immunolabeling of TRPV2 and TRPV3 and gene expression of TRPV1 in ETR. PPR shows enhanced immunoreactivity for TRPV2 and TRPV4 and increased TRPV2 gene expression (48). Zhou X. et al. identified that TPRV4 also interacts with transient receptor potential melastatin 8 (TRPM8) channels on immune cells or keratinocytes, which is strongly associated with itching in rosacea both in experimental and clinical settings (136).

## 5 Molecular targeted therapy in rosacea

Rosacea treatment primarily focuses on symptom management, including anti-inflammatory, immunomodulatory, microflora-regulating, and capillary dilation strategies. Common treatments include topical agents (azelaic acid, metronidazole, brimonidine, ivermectin, tacrolimus, pimecrolimus) and oral antibiotics (tetracycline, retinoids) (12, 137). However, increasing concerns over antibiotic resistance and impacts on skin flora indicate a pressing need for more effective and safer therapeutic alternatives (138). Emerging insights into the signaling pathways involved in rosacea mentioned above have led to the exploration of targeted therapies, aiming for improved efficacy and fewer side effects. **Table 1** presents current therapeutic targets and corresponding treatments for rosacea. However, the efficacy of these treatments remains challenging to assess and compare due to insufficient clinical studies.

**Table 1:** Summary of key signaling pathways and targeted treatments in rosacea.

Pathway	Targeted molecule	Example	References
LL37-related signaling pathways	TLR2, KLK5, LL-37, MMPs	Retinoids, Azelaic acid, Doxycycline, Carvedilol, Ivermectin	(139–143)
	mTORC1	Rapamycin, Celastrol	(10, 32)
	Th1/Th17-IL17	Secukinumab, Aspirin, Thalidomid	(69, 145, 146)
	VEGF	Topical dobesilate, Tranexamic acid	(97, 147)
JAK/STAT pathways	JAK2, STAT3	Tofacitinib	(11, 148)

### 5.1 Targeting TLR2-KLK5- LL37 and mTOR-related pathways

Targeting the TLR2-KLK5-LL37 pathway is currently a key strategy for the clinical treatment of rosacea. Retinoids, azelaic acid, and doxycycline modulate this pathway, reducing KLK5 and cathelicidin expression (139–141). Azelaic acid inhibits serine protease activity, and doxycycline limits KLK5 activity by inhibiting MMP9 (139, 142). Recent studies indicate that Carvedilol and Ivermectin modulate this pathway, contributing to their efficacy in rosacea treatment (143). A vitro study demonstrated that *ϵ*-Aminocaproic Acid (ACA) and Superoxide Dismutase 3 (SOD3) are effective in modulating the TLR2-related pathway (65, 144). Topical Rapamycin, an inhibitor of mTOR, has shown clinical effectiveness in treating rosacea. In a controlled study, 18 female rosacea patients were randomized to receive either a placebo or 0.4% FDA-approved rapamycin ointment. The results demonstrated that the group treated with rapamycin experienced significant clinical improvement compared to the placebo group, indicating the potential of mTORC1 inhibition as a therapeutic strategy in rosacea (10). Furthermore, Celastrol and Epigallocatechin-3-gallate (EGCG) also target mTOR-related pathways, exhibiting anti-inflammatory effects (31, 32).

### 5.2 Targeting Th1/Th17-IL17 in rosacea

The development of biological agents targeting specific cytokines offers a promising approach to treating rosacea. Approved antibodies, including those against IL-1*β* and IL-17, show potential as novel treatments. Specifically, secukinumab targeting IL-17, a monoclonal antibody primarily used in psoriasis, is under investigation for its effectiveness in treating rosacea. A trial involving 24 patients with papulopustular rosacea assessed the efficacy of secukinumab. The patients received 300 mg of secukinumab weekly for 5 weeks, then monthly for 2 months, the treatment led to significant improvement in papules and overall severity in 17 of the participants, along with enhanced quality of life (145). In addition, Aspirin and Thalidomide have shown potential in moderating Th1/Th17 immune responses, further supporting the strategy of targeting specific cytokine pathways in rosacea (69, 146).

### 5.3 Targeting VEGF in rosacea

VEGF inhibition has emerged as an effective strategy in rosacea treatment. Topical dobesilate, known for inhibiting angiogenic factors, has been shown effective in treating erythematotelangiectatic rosacea (147). Tranexamic acid, too, has shown efficacy in reducing microvessel density, VEGF expression, and associated inflammatory markers in rosacea patients (97). Additionally, the role of erythroid differentiation regulator 1 (Erdr1) in significantly inhibiting VEGF-mediated angiogenesis has been documented (73).

### 5.4 JAK/STAT pathway in rosacea

The JAK/STAT pathway plays a crucial role in the pathogenesis of rosacea. Oral tofacitinib, a JAK inhibitor, has demonstrated efficacy in mitigating facial erythema in rosacea. A clinical study with 21 rosacea patients revealed that 71.4% experienced a significant reduction in facial erythema following oral tofacitinib treatment (11). Furthermore, tofacitinib’s effectiveness in a case of steroid-induced rosacea underscores its potential, particularly in cases resistant to conventional therapies (148). Additionally, Artesunate has been identified as a promising agent in reducing inflammation through its action on the JAK2/STAT3 pathway (108).

## 6 Conclusion

This review highlights the complex signaling pathways involved in rosacea and the advancement of targeted therapies. The targeted modulation of the TLR2-KLK5-LL37 and mTOR pathways has shown significant efficacy in clinical settings. VEGF inhibitors have proven beneficial in treating erythematotelangiectatic rosacea. Biological agents, specifically monoclonal antibodies like secukinumab targeting IL-17, have been effective in treatment. The role of the JAK/STAT pathway in rosacea’s pathology is significant, with tofacitinib notably successful in reducing facial erythema. Despite these developments, research in targeted therapies for rosacea remains incomplete. Recognizing the complexity of rosacea, which involves multiple signaling pathways, is crucial for future advancements in treatment.
